# Fatty Acid Status and Its Relationship to Cognitive Decline and Homocysteine Levels in the Elderly

**DOI:** 10.3390/nu6093624

**Published:** 2014-09-12

**Authors:** Marília Baierle, Patrícia H. Vencato, Luiza Oldenburg, Suelen Bordignon, Murilo Zibetti, Clarissa M. Trentini, Marta M. M. F. Duarte, Juliana C. Veit, Sabrina Somacal, Tatiana Emanuelli, Tilman Grune, Nicolle Breusing, Solange C. Garcia

**Affiliations:** 1 Laboratory of Toxicology (LATOX), Department of Analysis, Pharmacy Faculty, Federal University of Rio Grande do Sul, Porto Alegre 90610000, RS, Brazil; E-Mails: mariliabaierle@yahoo.com.br (M.B.); patriciavencato@gmail.com (P.H.V.); luoldenburg@yahoo.com.br (L.O.); 2 Post-graduate Program in Pharmaceutical Sciences (PPGCF), Federal University of Rio Grande do Sul, Porto Alegre 90610000, RS, Brazil; 3 Institute of Psychology, Federal University of Rio Grande do Sul (UFRGS), Porto Alegre 90035003, RS, Brazil; E-Mails: su.suelen@gmail.com (S.B.); mugazibetti@gmail.com (M.Z.); clarissatrentini@terra.com.br (C.M.T.); 4 Department of Health Sciences, Lutheran University of Brazil, Santa Maria 97020001, RS, Brazil; E-Mail: duartmm@hotmail.com; 5 Department of Alimentary Technology and Science, Federal University of Santa Maria (UFSM), Santa Maria 97105900, RS, Brazil; E-Mails: juliana_veit@hotmail.com (J.C.V.); s_somacal@hotmail.com (S.S.); tatiemanuelli@gmail.com (T.E.); 6 Department of Nutritional Toxicology, Institute of Nutrition, Friedrich Schiller University Jena, Jena 07743, Germany; E-Mail: tilman.grune@uni-jena.de; 7 Department of Applied Nutritional Science and Dietetics, Institute of Nutritional Medicine, University of Hohenheim, Stuttgart 70593, Germany; E-Mail: breusing@uni-hohenheim.de

**Keywords:** aging, fatty acids, *n*-3 PUFA, cognitive decline, homocysteine, inflammation

## Abstract

Polyunsaturated fatty acids (PUFAs), especially the *n*-3 series, are known for their protective effects. Considering that cardiovascular diseases are risk factors for dementia, which is common at aging, the aim of this study was to evaluate whether fatty acid status in the elderly was associated with cognitive function and cardiovascular risk. Forty-five elderly persons (age ≥60 years) were included and divided into two groups based on their Mini-Mental Status Examination score adjusted for educational level: the case group (*n* = 12) and the control group (*n* = 33). Serum fatty acid composition, homocysteine (Hcy), hs-CRP, lipid profile and different cognitive domains were evaluated. The case group, characterized by reduced cognitive performance, showed higher levels of 14:0, 16:0, 16:1*n*-7 fatty acids and lower levels of 22:0, 24:1*n*-9, 22:6*n*-3 (DHA) and total PUFAs compared to the control group (*p* < 0.05). The *n*-6/*n*-3 ratio was elevated in both study groups, whereas alterations in Hcy, hs-CRP and lipid profile were observed in the case group. Cognitive function was positively associated with the 24:1*n*-9, DHA and total *n*-3 PUFAs, while 14:0, 16:0 and 16:1*n*-7 fatty acids, the *n*-6/*n*-3 ratio and Hcy were inversely associated. In addition, *n*-3 PUFAs, particularly DHA, were inversely associated with cardiovascular risk, assessed by Hcy levels in the elderly.

## 1. Introduction

The occurrence of chronic diseases increases as a function of age [1] and accounts for higher costs in public health as life expectancy increases around the world [2]. Cardiovascular and neurological diseases lead to a significant impairment in the activities of daily living [1]. Cognitive decline in particular acts adversely on functional independence, consequently decreasing quality of life [3].

Nutrition is commonly accepted as an environmental factor involved in the aging process that contributes to the prevention of chronic illnesses [4,5]. In this sense, epidemiological studies have demonstrated that polyunsaturated fatty acids (PUFAs) may affect several pathological conditions [6]. Thus, due to the inability of mammals to synthesize fatty acids with a double bond past the Δ-9 position [7], human intake of essential fatty acids from both the omega-3 and omega-6 series, such as α-linolenic acid (LNA, 18:3*n*-3) and linoleic acid (LA, 18:2*n*-6), respectively, is of extreme nutritional importance [8].

In the omega-3 series, eicosapentaenoic acid (EPA, 20:5*n*-3) and docosahexaenoic acid (DHA, 22:6*n*-3) are considered the most important [9]. *In vivo* studies using these PUFAs showed beneficial effects on brain functions, because they decreased motor disorders and memory dysfunction in rats [7], as well as improved episodic memory and learning functions in healthy adults [10]. In addition, they are associated with reduced cognitive decline during aging [4]. Brain tissue membranes are rich in *n*-3 PUFAs, which have anti-inflammatory, antioxidant, antiatherogenic, antiamyloid and neuroprotective properties [11]. *n*-3 PUFAs have also been associated with cardiovascular benefits, because they prevent cardiac arrhythmia, inhibit hepatic triglyceride synthesis, reduce platelet aggregation and, by modifying eicosanoid function, cause vascular relaxation and reduce inflammation [6,12]. Therefore, these actions represent an important mechanism to reduce the risk of cognitive decline, because cardiovascular diseases are risk factors for both vascular and degenerative dementia [11,13].

Elevated plasma homocysteine (Hcy) has been implicated as an independent risk factor for cardiovascular disease [14] and more recently for cognitive impairment [15]. Hcy is an endogenous sulfur amino acid, an intermediate in the synthesis of cysteine from methionine [12], which may increase vascular inflammation through various mechanisms [14]. However, the mechanism by which elevated Hcy levels promote vascular inflammation in neurodegenerative disorders, such as Alzheimer’s disease [16], is controversial. Moreover, placebo-controlled trials of *n*-3 PUFAs have demonstrated their ability to reduce Hcy levels [17]. Likewise, daily administration of oils rich in DHA and LA to rats significantly decreased the plasma Hcy concentration [18].

The potential effect of *n*-3 PUFAs on memory has been reported [3,19,20], being obtained exclusively from diet [3], and some reports demonstrated the positive effect of *n*-3 fatty acids supplementation on cognition, either by attenuating telomere shortening [19] or reducing depressive symptoms and the risk of progressing to dementia [20]. However, clinical trials have been inconclusive [21], and there is a gap on the role of different fatty acids, cardiovascular risk and their potential effects on cognitive function in elderly without neurological diseases. Given the increasingly fragile health of the brain and the cardiovascular system with age, the purpose of the present study was to evaluate the fatty acid status in the elderly with and without cognitive impairment in an attempt to establish potential relationships between these nutrients and cognitive function. Furthermore, this study investigated whether fatty acids are related to cardiovascular risk through homocysteine assessment in these populations.

## 2. Experimental Section

### 2.1. Subjects

Seventy elderly (aged ≥60 years) patients were recruited in Porto Alegre, Rio Grande do Sul, Brazil. Subjects were excluded from the study for the following criteria: difficult verbal communication, smokers, gastrectomized, diagnosed with cancer, psychiatric or neurological disease, taking parenteral nutrition, taking vitamin supplementation or who failed to collect samples or participate in any stage of the study. Based on these exclusion criteria, forty-five elderly subjects (15 male, 30 female) were enrolled.

The Mini-Mental Status Examination (MMSE) was performed [22], and cognitive impairment was defined as having an MMSE score lower than the cutoffs adjusted for educational level according to Kochhann *et al.* (2010) [1]. Regarding level of schooling, the cutoff values were 21 for the illiterate group, 22 for the low education group (1–5 years), 23 for the middle education group (6–11 years) and 24 for the high education group (≥12 years) [1]. According to this categorization, the case group was composed of 12 subjects: one illiterate subject, four subjects with low educational level and seven subjects with a middle educational level. The control group, in turn, consisted of 33 subjects: five, twenty and eight subjects with a low, middle and high educational level, respectively.

This study was approved by the ethics committees of the Federal University of Rio Grande do Sul (No. 15146) and the Clinical Hospital of Porto Alegre (No. 110171), and informed written consent was obtained from all subjects. Anthropometric measurements were determined. Body mass index (BMI) was calculated as body weight (kg) divided by the square of body height (m). Medical history, socioeconomic data and dietary intake data were obtained by questionnaire, and the Geriatric Depression Scale, short form (GDS), was also administered.

### 2.2. Laboratory Assays

Blood samples were drawn after 12-h overnight fasting by venipuncture in Vacutainer^®^ tubes without anticoagulant. They were centrifuged at 1500× *g* for 10 min at room temperature, and the serum was immediately used to assess the concentrations of total cholesterol, high density lipoprotein cholesterol (HDL-C) and triglyceride (TG) levels. The remaining serum was aliquoted and stored at −80 °C for determination of high-sensitivity C reactive protein (hs-CRP), homocysteine (Hcy) and fatty acids. The lipid profile was analyzed using a Labmax 240^®^ (Labtest Diagnostica, Lagoa Santa, Minas Gerais, Brazil) utilizing commercial kits. The low-density lipoprotein cholesterol (LDL-C) fraction was estimated by the Friedewald equation [23]. The index of coronary risk was calculated from the total cholesterol/HDL-C ratio. hs-CRP was measured by nephelometry (Dade Behring, Siemens, Erlangen, Bavaria, Germany). Hcy levels were assayed using a chemiluminescent enzyme immunoassay kit (Immulite2000, Los Angeles, CA, USA).

### 2.3. Fatty Acids Measurement

Fatty acids from serum were esterified and extracted using a one-step reaction [24], except that isooctane was used instead of benzene. Serum (100 µL) was mixed with 70 µg tricosanoic acid (internal standard), 2 mL methanol/isooctane (4:1, v/v) and 200 µL acetyl chloride and was incubated at 100 °C for 60 min; then 60 g·L^−1^ aqueous potassium carbonate containing 100 g·L^−1^ sodium chloride were added. The mixture was shaken for 10 min at room temperature and centrifuged at 1800× *g* for 5 min to obtain the isooctane phase containing the fatty acid methyl esters. Methylated fatty acids were analyzed by gas chromatography (Agilent Tech HP 6890N) with a capillary column (DB-23 60 m × 0.25 mm × 0.25 μm) and flame ionization detector (FID). The temperature of the injector port was set at 250 °C, and the carrier gas was nitrogen (0.9 mL·min^−1^). After injection (1 μL, split ratio 50:1), the oven temperature was held at 160 °C for 1 min, then increased to 240 °C at 4 °C·min^−1^ and held at this temperature for 9 min. Fatty acid methyl esters were identified by comparing the results to known standards (37-component FAME Mix, 22:5*n*-3 and PUFA No. 2 from Sigma, Saint Louis, MO, USA, and 22:5*n*-6 from NuChek Prep. Inc., Elysian, MN, USA). Fatty acids were expressed as a percentage of the total fatty acids identified.

### 2.4. Cognitive Function

In addition to the MMSE, the global cognitive function was assessed by a psychologist in individual interviews through the following tests adapted from the CERAD battery (Consortium to Establish a Registry for Alzheimer’s Disease): Verbal Fluency, Animal Category, Boston’s Naming Test (short version), Word List Memory, Delay Recall of Word List, Recognition Word List, Constructional Praxis, Delay Visual Memory, Trail Making Test (TMT) and Wechsler Adult Intelligence Scale 3rd Edition, Digit Subtest (WAIS-III). These cognitive screening instruments have been described previously [25]. Thus, a wide assessment of cognition regarding orientation, attention, concentration, language, spatial abilities, learning curve, perceptual and visuo-constructional skills and mental flexibility was possible, as well as evaluation of memory in its various aspects, such as the capacity for consolidation into long-term memory, immediate memory retention and working memory. High scores on all tests denoted better performance, except for the TMT, which is composed of Parts A and B in an increasing level of difficulty and for which the time spent to perform each part represents the score. In this case, cognitive ability is proportional to speed and inversely related to the time spent.

### 2.5. Statistical Analysis

Statistical analysis was conducted using SPSS (version 18). The results are presented as the mean ± standard error of mean (SEM). Categorical data were summarized as percentages, and comparisons among groups were performed with Fisher’s exact test. The Mann-Whitney *U*-test was used to determine significant differences in continuous variables between the groups. Correlation tests were performed according to Spearman’s rank. The power calculation was performed by WinPepi software (version 11.43). Univariate linear regression was applied to adjust for the influence of age on cognitive tests. Statistical significance was assumed at *p* < 0.05.

## 3. Results

A total of 45 subjects was divided into two groups according the MMSE performance, with (case group, *n* = 12) and without cognitive impairment (control group, *n* = 33). The baseline characteristics of study participants are described in Table 1. Significant differences were observed for total cholesterol, LDL-C, triglycerides and the total cholesterol/HDL-C ratio. These levels were increased in the case group compared to the control group (*p* < 0.05) and were also above the reference values [26]. However, there were no significant differences in age, hypertension, diabetes, GDS and BMI between the groups.

**Table 1:** Baseline characteristics of the elderly: the case group (with cognitive impairment) and control group (without cognitive impairment).

Parameter	Case (*n* = 12)	Control (*n* = 33)	Reference Values
Age (year)	78.58 ± 2.57	73.33 ± 1.36	
Male (%)	25.0	36.4	
Hypertension (%)	66.7	66.7	
Diabetes (%)	8.3	24.2	
GDS	4.00 ± 0.99	4.42 ± 0.54	
BMI (kg/m^2^)	25.38 ± 1.03	26.43 ± 1.01	
Total cholesterol (mg·dL^−1^)	243.75 ± 12.69 ^b^	201.06 ± 8.22	<200
LDL cholesterol (mg·dL^−1^)	154.33 ± 12.26 ^a^	121.88 ± 6.18	<130
HDL cholesterol (mg·dL^−1^)	47.08 ± 2.23	54.39 ± 3.12	>40
Total cholesterol/HDL-C ratio	5.33 ± 0.43 ^b^	3.92 ± 0.21	<5
Triglycerides (mg·dL^−1^)	212.50 ± 21.97 ^b^	123.73 ± 9.61	<150

Data are expressed as the mean and SEM or percentages. BMI, body mass index; GDS, geriatric depression scale; HDL, high-density lipoprotein; LDL, low-density lipoprotein. ^a^ *p* < 0.05; ^b^ *p* < 0.01 compared to the control group.

The results for the serum fatty acid status of the participants are shown in Table 2. The fatty acid profile in the case group compared to the control group was characterized by higher levels of the saturated fatty acids 14:0 and 16:0 and lower levels of 22:0 (*p* < 0.01). The levels found for the monounsaturated fatty acid (MUFA) 24:1*n*-9 were decreased (*p* < 0.01), while the levels of 16:1*n*-7 were increased (*p* < 0.05) in the case group compared to the control group. It was also found that there was a reduction in the levels of 22:6*n*-3 (DHA) and in the total polyunsaturated fatty acids compared to the control group (*p* < 0.05). The *n*-6/*n*-3 ratio was not significantly different between the groups (*p* > 0.05). Moreover, the dietary intake data obtained by questionnaire corroborated with the serum fatty acids levels (data not shown).

**Table 2:** Serum fatty acid composition of subjects expressed as the percentage (%) of total fatty acids identified.

Fatty Acid	Case (*n* = 12)	Control (*n* = 33)
14:0	0.98 ± 0.10 ^b^	0.67 ± 0.05
16:0	24.11 ± 0.65 ^b^	21.36 ± 0.38
18:0	9.44 ± 0.61	10.21 ± 0.36
22:0	1.05 ± 0.11 ^b^	1.66 ± 0.12
Σ Saturated	35.58 ± 1.03	33.91 ± 0.56
16:1*n*-7	2.14 ± 0.20 ^a^	1.60 ± 0.11
18:1*n*-9c	18.12 ± 0.69	16.99 ± 0.46
18:1*n*-7	1.43 ± 0.08	1.32 ± 0.05
24:1*n*-9	1.20 ± 0.09 ^b^	1.84 ± 0.14
Σ Monounsaturated	23.95 ± 1.17	22.58 ± 0.52
18:2*n*-6c	24.14 ± 1.17	25.70 ± 0.75
18:3*n*-6	0.55 ± 0.11	0.32 ± 0.07
20:2*n*-6	0.70 ± 0.23	0.77 ± 0.30
20:3*n*-6	2.66 ± 0.23	2.88 ± 0.18
20:4*n*-6	8.85 ± 0.48	9.79 ± 0.48
Σ *n*-6 Polyunsaturated	36.90 ± 0.95	39.46 ± 0.76
18:3*n*-3	0.62 ± 0.08	0.84 ± 0.20
20:5*n*-3	0.53 ± 0.09	0.51 ± 0.09
22:5*n*-3	0.84 ± 0.14	0.52 ± 0.10
22:6*n*-3	1.57 ± 0.14 ^a^	2.18 ± 0.18
Σ *n*-3 Polyunsaturated	3.57 ± 0.25	4.05 ± 0.36
Σ Polyunsaturated	40.47 ± 0.94 ^a^	43.51 ± 0.62
18:1*n*-9t	1.06 ± 0.56	0.85 ± 0.26
Σ trans	1.06 ± 0.56	0.85 ± 0.26
*n*-6/*n*-3 Ratio	11.05 ± 1.09	13.99 ± 2.69

Data are expressed as the mean ± SEM. ^a^ *p* < 0.05; ^b^ *p* < 0.01 compared to the control group (elderly subjects without cognitive impairment). The fatty acids, 4:0, 6:0, 8:0, 11:0, 12:0, 13:0, 14:1*n*-5, 15:0, 15:1*n*-5, 17:0, 17:1*n*-7, 18:2*n*-6t, 20:0, 20:1*n*-9, 21:0, 20:3*n*-3, 22:1*n*-9, 22:2*n*-6, 22:4*n*-6, 22:5-*n*6, 23:0, 24:0, were not detected in the samples.

The MMSE score ranged from eight to 22 points in the case group and 22 to 30 points in the control group, demonstrating impairment in the case group. Furthermore, other results of cognitive function from the different instruments applied also demonstrated cognitive impairment in case group compared with the control group results shown in Table 3, except in Recognition Word List and Trails Test B time, independent of age. Thus, it is possible to observe that of the 11 performed tests, only two did not demonstrate a significant difference. For tests, such as MMSE, Verbal Fluency, Word List Memory, Delay Recall of Word List and Trials Test A time, the case group presented a major decrease of about 45% compared to the control group.

**Table 3:** Cognitive performance of the studied groups in thedifferent applied instruments.

Instrument	Case (*n* = 12)	Control (*n* = 33)
MMSE	16.50 ± 1.24 ^b^	26.76 ± 0.38
Verbal Fluency	8.91 ± 0.99 ^b^	15.94 ± 1.04
Boston’s Naming Test	8.50 ± 0.74 ^b^	12.97 ± 0.38
Word List Memory	9.08 ± 1.41 ^b^	15.30 ± 0.84
Delay Recall of Word List	1.92 ± 0.67 ^a^	4.64 ± 0.47
Recognition Word List	6.10 ± 1.05	8.24 ± 0.40
Constructional Praxis	5.20 ± 0.73 ^b^	8.91 ± 0.30
Delay Visual Memory	1.75 ± 0.73 ^b^	6.41 ± 0.63
Trails Test A time (s)	167.00 ± 42.89 ^a^	85.50 ± 7.21
Trails Test B time (s)	268.33 ± 31.67	187.29 ± 16.04
WAIS-III Digits	7.17 ± 0.74 ^b^	10.45 ± 0.47

The values were adjusted for age and expressed as the mean ± SEM. MMSE, Mini-Mental Status Examination; WAIS-III, Wechsler Adult Intelligence Scale III Edition. ^a^ *p* < 0.05; ^b^ *p* < 0.01 compared to the control group (elderly subjects without cognitive impairment).

Table 4 shows the results between cognitive performance and fatty acids. Positive associations were found between different instruments for the evaluation of cognitive performance and the 24:1*n*-9, 22:6*n*-3 fatty acids and the total *n*-3 PUFAs, while saturated fatty acids (14:0; 16:0), 16:1*n*-7 and the *n*-6/*n*-3 ratio were inversely associated. However, no association was observed with 20:4*n*-6. Additionally, we evaluated only in the case group the potential associations between fatty acids *versus* cognitive performance. The results showed strong associations between the saturated fatty acid 14:0 *vs.* Trials Test A time (*r* = 0.812), the MUFA 16:1*n*-7 *vs.* Word List Memory (*r* = −0.587) and Delay Recall of Word List (*r* = −0.684) and the *n*-3 PUFA 22:6*n*-3 *vs.* Recognition Word List (*r* = 0.732) and Constructional Praxis (*r* = 0.667) (*p* < 0.05).

**Table 4:** Correlation coefficients (*r*) between cognitive performance (different applied instruments) and fatty acids (*n* = 45).

Instrument	14:0	16:0	16:1*n*-7	24:1*n*-9	20:4*n*-6	22:6*n*-3	Σ *n*-3 PUFAs	*n*-6/*n*-3 Ratio
MMSE	−0.597 ^b^	−0.396 ^b^	−0.405 ^b^	0.472 ^b^	0.137	0.546 ^b^	0.463 ^b^	−0.438 ^b^
Verbal Fluency	−0.544 ^b^	−0.230	−0.423 ^b^	0.367 ^a^	0.065	0.257	0.225	−0.242
Boston’s Naming Test	−0.499 ^b^	−0.229	−0.429 ^b^	0.543 ^b^	0.256	0.388 ^b^	0.224	−0.251
Word List Memory	−0.534 ^b^	−0.123	−0.387 ^b^	0.365 ^a^	0.262	0.417 ^b^	0.423 ^b^	−0.482 ^b^
Delay Recall of Word List	−0.427 ^b^	−0.187	−0.364 ^a^	0.231	0.251	0.447 ^b^	0.428 ^b^	−0.476 ^b^
Recognition Word List	−0.437 ^b^	−0.158	−0.430 ^b^	0.085	0.331	0.513 ^b^	0.437 ^b^	−0.401 ^b^
Constructional Praxis	−0.531 ^b^	−0.438 ^b^	−0.417 ^b^	0.326 ^a^	0.044	0.148	0.113	−0.113
Delay Visual Memory	−0.569 ^b^	−0.421 ^b^	−0.369 ^a^	0.330 ^a^	0.167	0.322 ^a^	0.291	−0.355 ^a^
Trials Test A time	0.545 ^b^	0.131	0.453 ^b^	−0.334 ^a^	−0.115	−0.436 ^b^	−0.476 ^b^	0.460 ^b^
Trials Test B time	0.424 ^a^	0.189	0.221	−0.361 ^a^	0.153	−0.335 ^a^	−0.387 ^a^	0.441 ^a^
WAIS-III Digits	−0.267	−0.166	−0.082	0.382 ^b^	−0.287	0.192	0.217	−0.287

MMSE, Mini-Mental Status Examination; WAIS-III, Wechsler Adult Intelligence Scale III Edition; PUFAs, polyunsaturated fatty acids. ^a^ *p* < 0.05; ^b^ *p* < 0.01.

**Figure 1:**
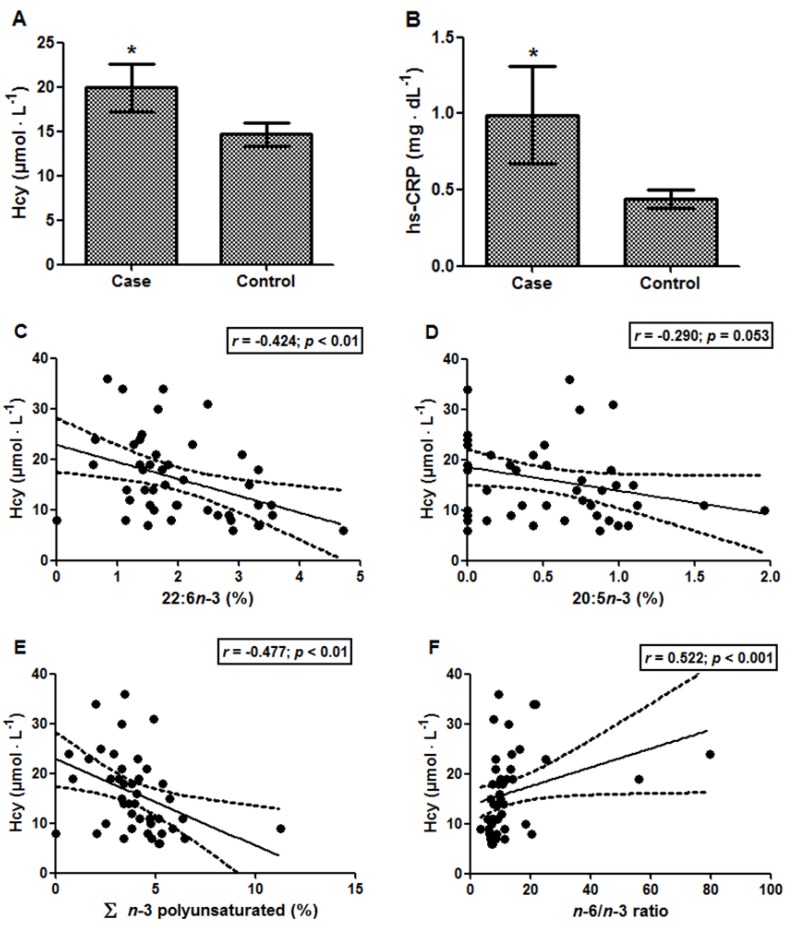
(**A**) Homocysteine (Hcy) and (**B**) hs-CRP levels in elderly subjects with (case) and without (control) cognitive impairment. Data are expressed as the mean ± SEM. * *p* < 0.05. Correlations between homocysteine *vs.*: (**C**) 22:6*n*-3; (**D**) 20:5*n*-3; (**E**) Σ *n*-3 polyunsaturated; and (**F**) the *n*-6/*n*-3 ratio. In each analysis, *n* = 45.

The results for Hcy and hs-CRP were significantly increased in the case group compared with the control group, Hcy levels being 19.92 ± 2.70 *vs.* 14.67 ± 1.28 µmol·L^−1^ (*p* < 0.05; Figure 1A) and hs-CRP levels being 0.99 ± 0.32 *vs.* 0.44 ± 0.06 mg·dL^−1^ (*p* < 0.05; Figure 1B), respectively. The Hcy levels of the case group were above the reference value (15 μmol·L^−1^) [26]. Moreover, a positive association was found between Hcy and total cholesterol/HDL-C ratio (*r* = 0.341; *p* < 0.05). Additionally, it was possible to observe that the increase in the Hcy levels was accompanied by a decrease in total *n*-3 PUFAs (*r* = −0.477; *p* < 0.01), especially 22:6*n*-3 (*r* = −0.424; *p* < 0.01) and 20:5*n*-3 (*r* = −0.290; *p* = 0.053) (Figure 1C–E). In contrast, the *n*-6/*n*-3 ratio was positively associated with Hcy levels (Figure 1F; *r* = 0.522; *p* < 0.001). Considering the reduced size of the elderly group, the power calculation was performed, and the following values were found: 83.4% (results of Figure 1C), 91.9% (results of Figure 1E) and 96.3% (results of Figure 1F). hs-CRP, in turn, presented only a tendency to correlate with 14:0, myristic acid (*r* = 0.289; *p* = 0.054). Moreover, Hcy levels were negatively associated with five instruments of cognitive performance (Table 5). However, no associations were demonstrated between hs-CRP and the cognitive performance of the 11 instruments assessed.

**Table 5:** Correlations between homocysteine (Hcy) levels *vs.* cognitive performance, using different instruments. In each analysis, *n* = 45.

Instrument	Hcy (μmol·L^−1^)
MMSE	−0.332 ^a^
Verbal Fluency	−0.153
Boston’s Naming Test	−0.180
Word List Memory	−0.370 ^a^
Delay Recall of Word List	−0.396 ^b^
Recognition Word List	−0.262
Constructional Praxis	−0.251
Delay Visual Memory	−0.190
Trials Test A time	0.317
Trials Test B time	0.383 ^a^
WAIS-III Digits	−0.306 ^a^

MMSE, Mini-Mental Status Examination; WAIS-III, Wechsler Adult Intelligence Scale III Edition; ^a^ *p* < 0.05; ^b^ *p* < 0.01.

## 4. Discussion

The Western diet has changed during the last few decades, increasing the intake of polyunsaturated fatty acids, but decreasing the intake of omega-3 essential fatty acids [5]. Although the sum of polyunsaturated fatty acids was lower in the case group compared to the control group, the *n*-6/*n*-3 ratio was high in both groups compared to the ratio found by Simon *et al.* (1995) [27] in men with (8.04) and without (7.21) coronary heart disease. According to the literature, a high ratio might indicate a predisposition for several chronic diseases [4]. Indeed, the *n*-6 and *n*-3 fatty acids have opposing effects, and their intake in the diet should be equivalent in healthy individuals [28]. Essentially, both *n*-6 and *n*-3 PUFAs are involved in the synthesis of eicosanoids [28], but those formed from arachidonic acid (AA, 20:4*n*-6), obtained from LA (*n*-6), have proinflammatory effects, whereas the mediators formed from EPA and DHA (*n*-3) have anti-inflammatory action [9,29]. Moreover, there is competition between the *n*-3 and *n*-6 fatty acid families for metabolism, because they share the same series of enzymes [28], so the conversion efficiency from LNA into EPA and DHA, which can occur endogenously through a series of metabolic reactions, is greatly reduced in the presence of excessive levels of LA [9], such as those observed in both of our study groups.

Among *n*-3 PUFAs, the DHA concentration is specifically high in the brain and is involved in memory formation, among other processes [8,30]. Therefore, its deficiency impairs normal neurological function [9]. In this study, lower DHA levels were found in the case group. Kishino *et al.* (2008) [31] found in a population of older adults 7.8% for *n*-3 PUFAs and 4.1% for DHA. These values are higher compared with the present study. However, Simon *et al.* (1995) [27] found higher *n*-3 PUFAs levels in healthy adults (5.38%).

The case group was characterized by a decline in cognitive function in almost all instruments used, showing a reduction in a wide range of cognitive aspects, such as orientation, retention or data logging, learning, memory and language. Indeed, these cognitive performance findings appear to indicate the existence of mild to moderate dementia in these groups, although the exclusion criteria ruled out those subjects with neurological disease.

In this scenario, correlations have been found between decreased levels of DHA and cognitive decline. Several mechanisms for these effects have been investigated *in vitro* and *in vivo*, as neuronal survival promoted by DHA [28,30,32]. Furthermore, strong positive associations were found between total *n*-3 PUFAs and better cognitive performance in the present study. The *n*-3 PUFA’s neuroprotective properties are not only due to its ability to antagonize the production of AA and proinflammatory eicosanoids [30]. Specific lipid messengers, such as the potent mediator neuroprotectin D1, synthesized from DHA, exert anti-inflammatory actions and attenuate apoptotic processes by oxidative stress [30,33]. Of note, both chronic inflammation and oxidative stress have been demonstrated in patients with age-related cognitive impairment and neurodegenerative diseases [34,35].

Our findings are consistent with those previously reported by Akbar *et al.* (2005) [32], who showed the specific effect of *n*-3 PUFAs, such as DHA, in preventing oxidative stress-induced apoptosis of neuroblastoma 2A cells. In addition, our results suggest a beneficial effect of 24:1*n*-9 (nervonic acid) on different domains of cognition. Interestingly, the present work found correlations between this monounsaturated fatty acid and tests that assessed the attention and language domains, but not exclusively. A previous study of the blood fatty acid composition in children with attention-deficit hyperactivity disorder (ADHD) showed that nervonic acid levels were significantly lower than in normal children [36]. However, this requires further investigation, because ADHD and dyslexia in adults were associated with elevated nervonic acid in Project Adult Dyslexia and Attention Deficit Disorder in Finland (Project DyAdd) [37]. In parallel, a prospective observational study that assessed dietary monounsaturated fat intake using a food frequency questionnaire demonstrated their association with reduced cognitive decline in older women [38]. Beyond their involvement in inflammatory pathways [29], MUFAs appear to have antioxidant effects [39], and although there is some evidence that they favorably alter serum lipids, ambiguous results remain with respect to their effects on the risk of chronic disease [40]. Not all fatty acids within a group function similarly [41], and this corroborates the negative correlations found between another MUFA, palmitoleic acid (16:1*n*-7), and cognitive performance. These findings are in line with previous data showing increased palmitoleic acid levels in the brain associated with cognitive impairments [42]. Particularly, this MUFA has already been positively associated with markers of inflammation [41], which may be underlying such effects.

Thus, with the exception of nervonic and palmitoleic acids, the levels of other MUFAs evaluated in our case group did not differ from those of the control group. However, myristic (14:0) and palmitic acid (16:0), which are both saturated, were present in higher levels in the case group and were associated with cognitive decline. Hussain *et al.* (2013) [43] showed in their review evidence that palmitic acid may trigger the production of the Aβ peptide and the formation of amyloid plaques, the main neuropathological hallmark of Alzheimer’s disease, which can be suppressed by *n*-3 PUFAs. However, studies of the apparent relationship between myristic acid and cognitive function are scarce. In fact, saturated fats have been thought to raise plasma total cholesterol and LDL-C by downregulating the hepatic LDL receptor system, which decreases the clearance of this cholesterol from the bloodstream, although other mechanisms may be possible [44]. This corroborates the fact that the case group presented higher lipid profiles. LDL in the arteries can be a target of oxidative stress, becoming oxidized and initiating the atherogenic process, which leads to blocked blood flow and, therefore, to heart attack and cerebrovascular accident, popularly known as stroke [44]. In this line, these findings of elevated lipids reinforce the predisposition of these elderly for cognitive impairment due to this comorbidity. Cognitive decline was also associated with a high *n*-6/*n*-3 ratio. In this context, eicosanoids from AA are biologically active in small quantities, but when formed in large amounts, as discussed earlier, they contribute to the formation of thrombus and atheromas, increasing the potential for the development of brain injury as ischemic cerebrovascular disease [29].

Taken together, the present results are in agreement with a cross-sectional study that found an association between increased *n*-3 PUFA intake and improvements in cognitive function [4]. However, Kim *et al.* (2010) [11] showed an association between lower risk of dementia in elderly Korean subjects with higher levels of LNA, but not DHA, EPA and total *n*-3 PUFA levels. Nevertheless, both studies assessed cognitive decline exclusively with the MMSE, which is likely not as sensitive as the neuropsychological test battery used in this study.

In this line, our results corroborated those of Milte and collaborators [3], which also demonstrated that *n*-3 and *n*-6 PUFAs, obtained exclusively from diet, present positive and negative effects, respectively, on cognition in the elderly. Thus, the modifications in PUFA intakes could affect, in fact, the memory in adults. In the present study, a strong association between DHA and tasks of memory (Recognition Word List and Constructional Praxis) was found in the case group, whereas in both groups, besides such associations, moderate associations were found between *n*-3 PUFAs, especially DHA, and attention and mental flexibility evaluated by the Trials tests. However, according to Milte and collaborators and other authors [3,20], the depression symptoms were postulated as modulators to poor cognition and memory associated with poor intakes of *n*-3 PUFAs. On the other hand, an association among GDS scores, cognition and intakes of PUFAs seem controversial, e.g., in our results, GDS scores were similar to both groups and, according to the Geriatric Depression Scale (GDS) [45,46], they could be considered without evidence of depression. However, there is limitation related to the small sample size of the case group in the present study. Therefore, further studies in this line will be important.

Moreover, in the present study, elderly subjects with cognitive deficit (case group) displayed increased Hcy levels. We also observed a relationship between Hcy and the total cholesterol/HDL-C ratio, which has been considered a valuable marker in determining coronary heart disease risk [40]. The vasculopathic effects of Hcy are mediated via decreased expression of apo A-I, which results in a decrease in HDL anti-inflammatory activity [47]. Accordingly, it is possible to predict greater vulnerability to vascular diseases in this population through evidence-based risk factors.

Dietary factors have been shown to influence the pathogenesis of vascular disease [48]. In this context, elevated levels of Hcy were inversely associated with total *n*-3 PUFAs, especially DHA. Additionally, a relationship was observed between higher Hcy levels and decreased EPA. The present finding is consistent with previous data reported in a Japanese study [12]. In addition, the *n*-6/*n*-3 ratio was positively associated with serum Hcy concentration; however, the mechanisms by which PUFAs decrease Hcy are not yet well understood [17]. Hcy concentrations are partly determined by genetic factors [12], thus animal studies suggest that mRNA expression of key genes encoding enzymes involved in homocysteine metabolism, such as methionine adenosyltransferase (MAT), cystathionine-γ-lyase (CSE) and 5-methyltetrahydrofolate reductase (MTHFR), could be regulated by *n*-3 PUFAs [18]. One of the pathways upregulates CSE activity and CSE mRNA expression. CSE catalyzes the conversion of cystathionine into cysteine and is the rate-limiting enzyme in the synthesis of cysteine from Hcy [18]. Thus, upregulated CSE mRNA expression expedites the degradation of cystathionine, causing decreased Hcy [18]. However, further studies are needed once it has been demonstrated that each PUFA has specific effects on the mRNA expression of such genes [18], and genetic variants may also influence these effects in humans [17].

Additionally, the hs-CRP levels in the case group differed from that of the control group. Increased hs-CRP, an acute-phase protein produced in response to inflammatory stimuli [48], was consistent with the observed fatty acid profile. Nevertheless, the level of hs-CRP unexpectedly did not correlate with the level of any fatty acid, despite its role in inflammation. It only exhibited a tendency to correlate with myristic acid. There are insufficient studies using diets rich in saturated fats to alter the immune response [48], and the limited evidence precludes conclusions about the potential proinflammatory effects of myristic acid [44].

The deleterious effects of Hcy are not limited to vascular diseases. Thus, evidence supports a role for high Hcy levels in dementia [15]. However, after the development of Alzheimer’s disease with mild to moderate cognitive impairment, even lowering Hcy levels with supplementation of vitamin B6 and B12, this cognitive framework is not reversed [49]. We also found an association between increased Hcy and reduced cognitive abilities. Hcy contributes to increased vascular inflammation in part via oxidative stress, a process to which the brain is especially vulnerable due to its large rate of oxygen consumption [14]. Hcy disrupts antioxidant defenses (enzymatic and non-enzymatic), and it induces the generation of reactive species by activation of glutamatergic receptors and/or by autoxidation to homocysteine and other disulfides [14], which may be a reason for the present finding.

To our knowledge, there is no study that relates the serum fatty acid composition to cognitive function and Hcy levels simultaneously. Blood levels of fatty acids reflect not only diet, but also their absorption and metabolism [11]. The assessment of multiple domains of cognition allowed for more detailed characterization of the cognitive effects, because different fats have differential effects on specific elements of cognitive function. On the other hand, a limitation of our study was the small number of patients with cognitive decline in the sample. However, by performing the power calculation, it was observed that the associations between Hcy and fatty acids allow suggesting the existence of a functional link among these parameters.

## 5. Conclusions

A limitation of this study was its conduction with a case group of a small sample size; therefore, further scientific work in this line with larger samples is required to assess the role of each fatty acid on cognitive function. However, in summary, decreased levels of *n*-3 PUFAs, especially DHA, and increased myristic and palmitic acids, which are both saturated, appear to be associated with cognitive decline. Lower levels of *n*-3 PUFAs may also be related to increased Hcy levels, which leads to vascular disease and, thus, may be involved in the risk of cognitive decline and other brain dysfunctions. Therefore, balanced intake of both *n*-3 and *n*-6 fatty acids is essential for health by avoiding a harmful permanent proinflammatory state.

## References

[1] Kochhann R., Varela J.S., Lisboa C.S.M., Chaves M.L.F. (2010). Review of cutoff points adjusted for schooling in a large Southern Brazilian sample. Dement. Neuropsychol..

[2] UNFPA and HelpAge International (2012). Ageing in the Twenty-First Century: A Celebration and a Challenge. http://unfpa.org/ageingreport.

[3] Milte C.M., Sinn N., Street S.J., Buckley J.D., Coates A.M., Howe P.R.C. (2011). Erythrocyte polyunsaturated fatty acid status, memory, cognition and mood in older adults with mild cognitive impairment and healthy controls. Prostaglandins Leukot. Essent. Fatty Acids.

[4] Velho S., Marques-Vidal P., Baptista F., Camilo M.E. (2008). Dietary intake adequacy and cognitive function in free-living active elderly: A cross-sectional and short-term prospective study. Clin. Nutr..

[5] Ramirez-Ramirez V., Macias-Islas M.A., Ortiz G.G., Pacheco-Moises F., Torres-Sanchez E.D., Sorto-Gomez T.E., Cruz-Ramos J.A., Orozco-Aviña G., de la Rosa A.J.C. (2013). Efficacy of fish oil on serum of TNF alpha, IL-1 beta, and IL-6 oxidative stress markers in multiple sclerosis treated with interferon beta-1b. Oxid. Med. Cell. Longev..

[6] Ubeda N., Achon M., Varela-Moreiras G. (2012). Omega 3 fatty acids in the elderly. Br. J. Nutr..

[7] Barcelos R.C., Benvegnu D.M., Boufleur N., Reckziegel P., Müller L.G., Pase C., Emanuelli T., Bürger M.E. (2010). Effects of omega-3 essential fatty acids (omega-3 EFAs) on motor disorders and memory dysfunction typical neuroleptic-induced: Behavioral and biochemical parameter. Neurotox. Res..

[8] Trevizol F., Benvegnu D.M., Barcelos R.C., Boufleur N., Dolci G.S., Müller L.G., Pase C.S., Reckziegel P., Dias V.T., Segat H. (2011). Comparative study between *n*-6, trans and *n*-3 fatty acids on repeated amphetamine exposure: A possible factor for the development of mania. Pharmacol. Biochem. Behav..

[9] Calviello G., Su H.M., Weylandt K.H., Fasano E., Serini S., Cittadini A. (2013). Experimental evidence of omega-3 polyunsaturated fatty acid modulation of inflammatory cytokines and bioactive lipid mediators: Their potential role in inflammatory, neurodegenerative, and neoplastic diseases. Biomed. Res. Int..

[10] Yurko-Mauro K., McCarthy D., Rom D., Nelson E.B., Ryan A.S., Blackwell A., Salem N., Stedman M. (2010). Beneficial effects of docosahexaenoic acid on cognition in age-related cognitive decline. Alzheimers Dement..

[11] Kim M., Nam J.H., Oh D.H., Park Y. (2010). Erythrocyte alpha-linolenic acid is associated with the risk for mild dementia in Korean elderly. Nutr. Res..

[12] Kume A., Kurotani K., Sato M., Ejima Y., Pham N.M., Nanri A., Kuwahara K., Mizoue T. (2013). Polyunsaturated fatty acids in serum and homocysteine concentrations in Japanese men and women: A cross-sectional study. Nutr. Metab..

[13] Laurin D., Verreault R., Lindsay J., Dewailly E., Holub B.J. (2003). Omega-3 fatty acids and risk of cognitive impairment and dementia. J. Alzheimers Dis..

[14] Zanin R.F., Campesato L.F., Braganhol E., Schetinger M.R.C., Wyse A.T.S., Battastini A.M.O. (2010). Homocysteine decreases extracellular nucleotide hydrolysis in rat platelets. Thromb. Res..

[15] Ford A.H., Flicker L., Alfonso H., Hankey G.J., Norman P.E., van Bockxmeer F.M., Almeida O.P. (2012). Plasma homocysteine and MTHFRC677T polymorphism as risk factors for incident dementia. J. Neurol. Neurosurg. Psychiatry.

[16] Veryard L., Jones E., Weaving G., Smith E., Cheek L., Wickramasinghe A., Tabet N. (2013). Pro-inflammatory cytokines IL-1beta and TNF-alpha are not associated with plasma homocysteine concentration in Alzheimer’s disease. Curr. Alzheimer Res..

[17] Huang T., Zheng J., Chen Y., Yang B., Wahlqvist M.L., Li D. (2011). High consumption of omega-3 polyunsaturated fatty acids decrease plasma homocysteine: A meta-analysis of randomized, placebo-controlled trials. Nutrition.

[18] Huang T., Hu X., Khan N., Yang J., Li D. (2013). Effect of polyunsaturated fatty acids on homocysteine metabolism through regulating the gene expressions involved in methionine metabolism. Sci. World J..

[19] O’Callaghan N., Parletta N., Milte C.M., Benassi-Evans B., Fenech M., Howe P.R.C. (2013). Telomere shortening in elderly individuals with mild cognitive impairment may be attenuated with ω-3 fatty acid supplementation: A randomized controlled pilot study. Nutrition.

[20] Sinn N., Milte C.M., Street S.J., Buckley J.D., Coates A.M., Petkov J., Howe P.R.C. (2012). Effects of *n*-3 fatty acids, EPAv. DHA, on depressive symptoms, quality of life, memory and executive function in older adults with mild cognitive impairment: A 6-month randomised controlled trial. Br. J. Nutr..

[21] Sinn N., Milte C., Howe P.R.C. (2010). Oiling the brain: A review of randomized controlled trials of omega-3 fatty acids in psychopathology across the lifespan. Nutrients.

[22] Folstein M.F., Folstein S.E., McHugh P.R. (1975). “Mini-mental state”. A practical method for grading the cognitive state of patients for the clinician. J. Psychiatr. Res..

[23] Friedewald W.T., Levy R.I., Fredrickson D.S. (1972). Estimation of the concentration of low-density lipoprotein cholesterol in plasma, without use of the preparative ultracentrifuge. Clin. Chem..

[24] Lepage G., Roy C.C. (1986). Direct transesterification of all classes of lipids in a one-step reaction. J. Lipid Res..

[25] Morris J.C., Heyman A., Mohs R.C., Hughs J.P., van Belle G., Fillenbaum G., Mellits E.D., Clark C., CERAD investigators (1989). The consortium to establish a registry for Alzheimer’s disease (CERAD). Part I. Clinical and neuropsychological assessment of Alzheimer’s disease. Neurology.

[26] Burtis C., Ashwood E., Bruns D.E. (2008). Tietz Fundamentals of Clinical Chemistry.

[27] Simon J.A., Hodgkins M.L., Browner W.S., Neuhaus J.M., Bernert J.T., Hulley S.B. (1995). Serum fatty acids and the risk of coronary heart disease. Am. J. Epidemiol..

[28] Schmitz G., Ecker J. (2008). The opposing effects of *n*-3 and *n*-6 fatty acids. Prog. Lipid Res..

[29] Simopoulos A.P. (1999). Essential fatty acids in health and chronic disease. Am. J. Clin. Nutr..

[30] Bazan N.G. (2007). Omega-3 fatty acids, pro-inflammatory signaling and neuroprotection. Curr. Opin. Clin. Nutr. Metab. Care.

[31] Kishino T., Watanabe K., Urata T., Takano U., Uemura T., Nishikawa K., Mine Y., Matsumoto M., Ohtsuka K., Ohnishi H. (2008). Visceral fat thickness in overweight men correlates with alterations in serum fatty acid composition. Clin. Chim. Acta.

[32] Akbar M., Calderon F., Wen Z., Kim H.Y. (2005). Docosahexaenoic acid: A positive modulator of Akt signaling in neuronal survival. Proc. Natl. Acad. Sci. USA.

[33] Bazan N.G. (2006). The onset of brain injury and neurodegeneration triggers the synthesis of docosanoid neuroprotective signaling. Cell. Mol. Neurobiol..

[34] Cervellati C., Cremonini E., Bosi C., Magon S., Zurlo A., Bergamini C.M., Zuliani G. (2013). Systemic oxidative stress in older patients with mild cognitive impairment or late onset Alzheimer’s disease. Curr. Alzheimer Res..

[35] Rothenburg L.S., Herrmann N., Swardfager W., Black S.E., Tennen G., Kiss A., Gladstone D.J., Ween J., Snaiderman A., Lanctôt K.L. (2010). The relationship between inflammatory markers and post stroke cognitive impairment. J. Geriatr. Psychiatry Neurol..

[36] Chen J.R., Hsu S.F., Hsu C.D., Hwang L.H., Yang S.C. (2004). Dietary patterns and blood fatty acid composition in children with attention-deficit hyperactivity disorder in Taiwan. J. Nutr. Biochem..

[37] Laasonen M., Hokkanen L., Leppamaki S., Tani P., Erkkila A.T. (2009). Project DyAdd: Fatty acids in adult dyslexia, ADHD, and their comorbid combination. Prostaglandins Leukot. Essent. Fatty Acids.

[38] Naqvi A.Z., Harty B., Mukamal K.J., Stoddard A.M., Vitolins M., Dunn J.E. (2011). Monounsaturated, trans, and saturated fatty acids and cognitive decline in women. J. Am. Geriatr. Soc..

[39] Briante R., Febbraio F., Nucci R. (2003). Antioxidant properties of low molecular weight phenols present in the Mediterranean diet. J. Agric. Food Chem..

[40] Schwingshackl L., Hoffmann G. (2012). Monounsaturated fatty acids and risk of cardiovascular disease: Synopsis of the evidence available from systematic reviews and meta-analyses. Nutrients.

[41] Perreault M., Roke K., Badawi A., Nielsen D.E., Abdelmagid S.A., el-Sohemy A., Ma D.W.L., Mutch D.M. (2014). Plasma levels of 14:0, 16:0, 16:1*n*-7, and 20:3*n*-6 are positively associated, but 18:0 and 18:2*n*-6 are inversely associated with markers of inflammation in young healthy adults. Lipids.

[42] Snigdha S., Astarita G., Piomelli D., Cotman C.W. (2012). Effects of diet and behavioral enrichment on free fatty acids in the aged canine brain. Neuroscience.

[43] Hussain G., Schmitt F., Loeffler J.P., de Aguilar J.L. (2013). Fatting the brain: A brief of recent research. Front. Cell. Neurosci..

[44] Micha R., Mozaffarian D. (2010). Saturated fat and cardiometabolic risk factors, coronary heart disease, stroke, and diabetes: A fresh look at the evidence. Lipids.

[45] Sheikh J.I., Yesavage J.A. (1986). Geriatric Depression Scale (GDS): Recent evidence and development of a shorter version. Clin. Gerontol..

[46] Marc L.G., Raue P.J., Bruce M.L. (2008). Screening performance of the geriatric depression scale (GDS-15) in a diverse elderly home care population. Am. J. Geriatr. Psychiatry.

[47] Devlin A.M., Lentz S.R. (2006). ApoA-I: A missing link between homocysteine and lipid metabolism?. Circ. Res..

[48] Voon P.T., Ng T.K., Lee V.K., Nesaretnam K. (2011). Diets high in palmitic acid (16:0), lauric and myristic acids (12:0 + 14:0), or oleic acid (18:1) do not alter postprandial or fasting plasma homocysteine and inflammatory markers in healthy Malaysian adults. Am. J. Clin. Nutr..

[49] Aisen P.S., Schneider L.S., Sano M., Diaz-Arrastia R., van Dyck C.H., Weiner M.F., Bottiglieri T., Jin S., Stokes K.T., Thomas R.G. (2008). High-dose B vitamin supplementation and cognitive decline in Alzheimer disease: A randomized controlled trial. JAMA.

